# Postmortem Interval Estimation Based on the Developmental Patterns of *Hemilucilia segmentaria* (Fabricius) (Diptera, Calliphoridae) and *Peckia* (*Euboettcheria*) *anguilla* (Curran & Walley) (Diptera, Sarcophagidae): a Case in Southeastern Brazil

**DOI:** 10.1007/s13744-026-01368-9

**Published:** 2026-03-18

**Authors:** Carina Mara Souza, Vinícius da Costa-Silva, Aline Marrara Prado, Andre Gardelino Savino, Patricia Jacqueline Thyssen

**Affiliations:** 1 Laboratory of Integrative Entomology, Department of Animal Biology, IB, Universidade Estadual de Campinas (UNICAMP), Campinas, São Paulo State Brazil; 2 Invertebrate Biosystematics and Conservation Group, Department of Zoology & Entomology, University of Pretoria, Pretoria, South Africa; 3 Forensic Police of São Paulo State, Superintendência da Polícia Técnico-Científica, Jundiaí, São Paulo State Brazil

**Keywords:** Necrophagous species, Bionomics, Period of insect activity, Forensic entomology

## Abstract

Decaying corpses constitute a relevant source of food resources for a wide range of insects classified as necrophagous. Several blowflies (Diptera: Calliphoridae) and flesh flies (Diptera: Sarcophagidae) species are necrophagous and, for this reason, comprise two of the most relevant families of insects in forensics. Obtaining reliable postmortem interval (PMI) estimates based on entomological traces is of great importance in investigative processes. We present a case report of PMI estimation based on entomological evidence collected in a young woman’s corpse found in a wild area from southeastern Brazil, using two Diptera species: *Hemilucilia segmentaria* (Fabricius) (Calliphoridae) and *Peckia* (*Euboettcheria*) *anguilla* (Curran & Walley) (Sarcophagidae). Additionally, we report for the first time *P.* (*E.*) *anguilla* rearing in a decaying corpse.

## Introduction

Decaying corpses represent a transient and ephemeral food source for several organisms, among which muscoid Diptera stand out, both in terms of abundance and diversity (e.g., Carvalho et al. 2000; 2004; Thyssen et al. 2018). Within the forensic scope, information provided by insects and other arthropods, combined with other expert procedures, can be valuable for the progress or conclusion of an investigative process (Catts and Goff 1992; Thyssen 2011; Lutz et al. 2021). This logic includes the estimation of the postmortem interval (PMI), which can be assessed based on knowledge of the period of insect activity (PIA) (Amendt et al. 2007), as it has usually been reported in the Neotropical region (e.g. Oliva 2001; Barreto et al. 2002; Oliveira-Costa and Mello-Patiu 2004; Pujol-Luz et al. 2006; Bermúdez and Pachar 2010; Ortloff-Trautmann et al. 2013; Souza et al. 2014; Vasconcelos et al. 2014; Gaedke and Mouga 2017; 2024; Pérez et al. 2017; Ramos et al. 2018; Thyssen et al. 2018; Corrêa et al. 2019; García-Ruilova et al. 2020; Lira et al. 2020b; Meira et al. 2020; Amat et al. 2021; Ramos-Pastrana et al. 2021; Siri et al 2021; Barros et al. 2024; Botteon et al. 2024a).

PIA has most often been estimated by considering the growth rate as a function of the thermal requirements inherent to each insect species (e.g. Nassu et al. 2014; Vairo et al. 2015; Grella et al. 2024) and calculated from a temperature-dependent linear developmental model (Ikemoto and Takai 2000). Alternatively, PIA can be assessed using morphometric data, such as changes in body length and mass resulting from the development of fly larvae, for example, as a function of the temperature to which a decaying corpse is exposed (e.g. Thyssen et al. 2012; 2014; 2023). In most cases, PIA accurately and reliably represents PMI, particularly when insects colonize the corpse shortly after death (Amendt et al. 2007). However, it is essential to highlight that any methods based on biological or ecological parameters are not applicable without the correct identification of the entomofauna through morphology, genes, and chemical features, among others (e.g. Thyssen et al. 2005; Braga et al. 2016; Prado et al. 2023).

Currently, there are approximately 70 and 950 recorded species of dipterans belonging to the families Calliphoridae and Sarcophagidae, respectively, in the Neotropics (Kosmann et al. 2013; Marinho et al. 2017; Pape 2021). Although a wide diversity of feeding habits and trophic specializations has been reported among representatives of both families (Ferrar, 1987), saprophagy appears to be a habit predominantly associated with most of their species (Madeira-Ott et al. 2022; Cardoso et al. 2025). For this reason, blowflies and flesh flies are among the most frequent colonizers of decaying corpses (Marcondes and Thyssen 2017).

Several efforts have been made to spread and highlight the importance and applicability of using entomological evidence to aid or conclude a case under investigation (Oliveira-Costa and Lopes 2000, Oliveira-Costa and Mello-Patiu 2004; Pujol-Luz et al. 2006; Poli-Neto et al. 2009; Kosmann et al. 2011; Thyssen et al. 2012; Souza et al. 2013; Souza et al. 2014; Vasconcelos et al. 2019; Vairo et al. 2015; 2017; Pérez et al. 2017; Ramos-Pastrana and Wolff 2017; Thyssen et al. 2018; Lira et al. 2020b; Acosta et al. 2021; Eulalio et al. 2021; Ramos-Pastrana et al. 2022; Battán-Horenstein et al. 2023; Botteon et al. 2024a; 2024b; 2024c). Considering the relevance of obtaining reliable PMI estimates, we report a case in which entomological traces pointed out the post-mortem interval of a young woman, whose corpse was found in a wild environment in southeastern Brazil. Additionally, the first report of *Peckia* (*Euboettcheria*) *anguilla* (Curran & Walley) (Diptera, Sarcophagidae) rearing in a decaying corpse is presented.

## Material and methods

Third-instar fly larvae were first collected at the scene where the corpse was found. A second collection of immature insects was also performed during the necropsy, at the Forensic Medical Institute, approximately eight hours after the first collection. Some collected specimens were stored in vials containing 70% ethanol, while others were stored in Kahle's solution (prepared as proposed by Borror et al. 1981) to ensure better preservation of the material for subsequent morphological examination.

Internal and external anatomical characters of the larvae were examined and photographed using a Carl Zeiss™ Discovery V. 12 stereomicroscope with an Axiocam MRc5 image capture system and Zen Pro 2012™ software with depth focus. The cephaloskeleton, an internal structure, was accessed by dissection and clarification in a 10% potassium hydroxide (KOH) solution, as proposed by Sukontason et al. (2004). Specific identification was carried out using taxonomic keys and descriptions (Thyssen and Linhares 2007; Thyssen 2010; Grella 2016; Prado et al. 2023).

Morphometric data of the larvae, such as length and body mass, were measured using Zen Pro™ 2012 software and a Bel™ analytical balance (model MARK M214A with a minimum sensitivity of 0.001 g), respectively. Meteorological data (daily averages for temperature, humidity, and accumulated rainfall) were obtained from the Brazilian National Institute for Space Research (INPE 2025).

## Results

### Case report

**Fig. 1:**
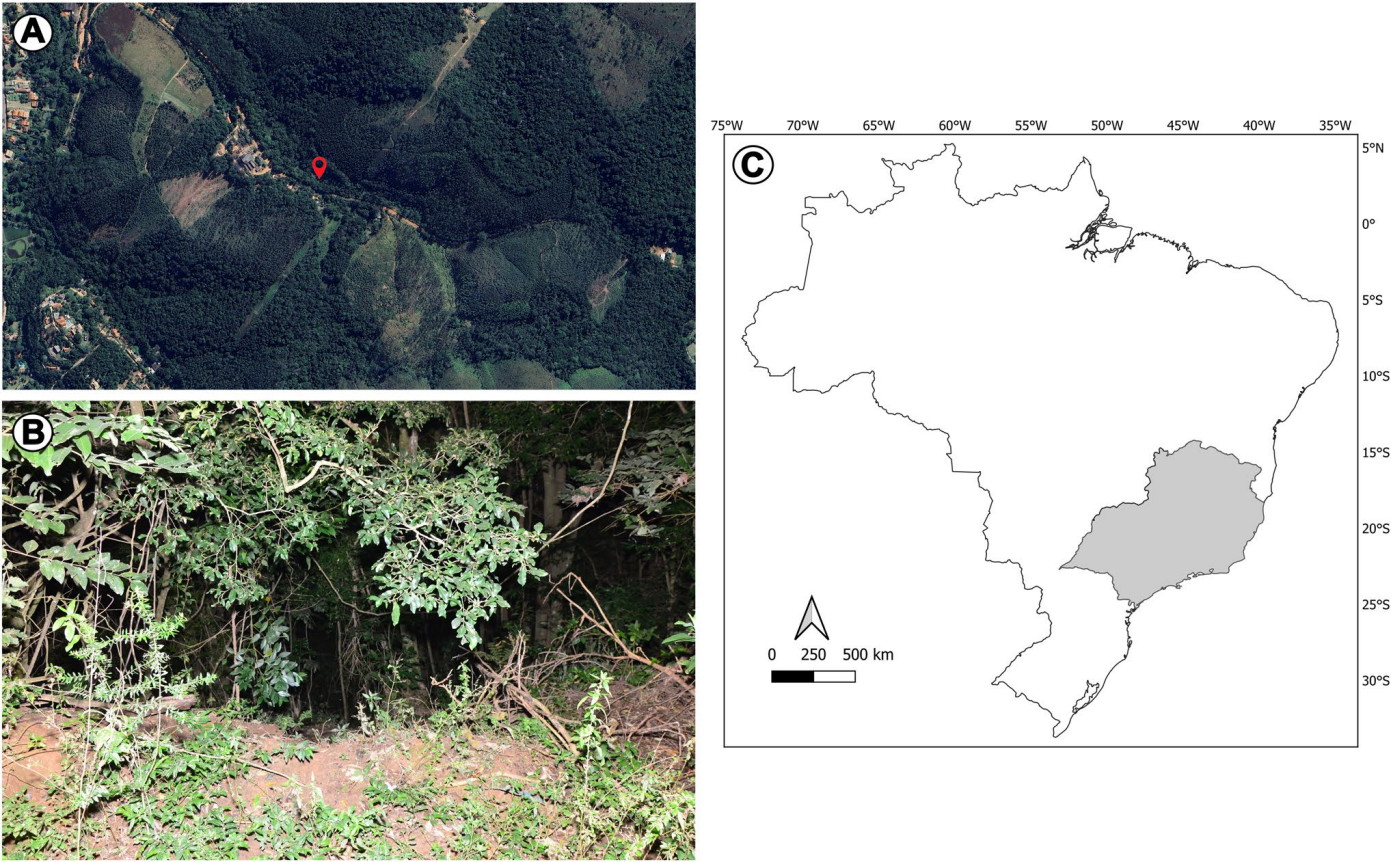
Location where the corpse was found. In: (**A**) panoramic view of the area (modified from Google Maps™) (**B**) vegetation composition characteristic of the Atlantic Forest biome; (**C**) the incident occurred in the southeastern region of Brazil, in gray (coordinates have been suppressed to prevent the exact identification of the events and associated victims)

An 18-year-old woman's corpse was found on a trail in a wilderness area about 1,000 m from a small residential village in southeastern Brazil. With a typical Atlantic Forest biome profile, the area is primarily covered by native plant species, whose heights range from 12 to 20 m, interspersed with a few areas of shorter tree species (~ 2 to 6 m tall) and some low infestation rates of *Brachiaria decumbens* (Fig. 1).

**Fig. 2:**
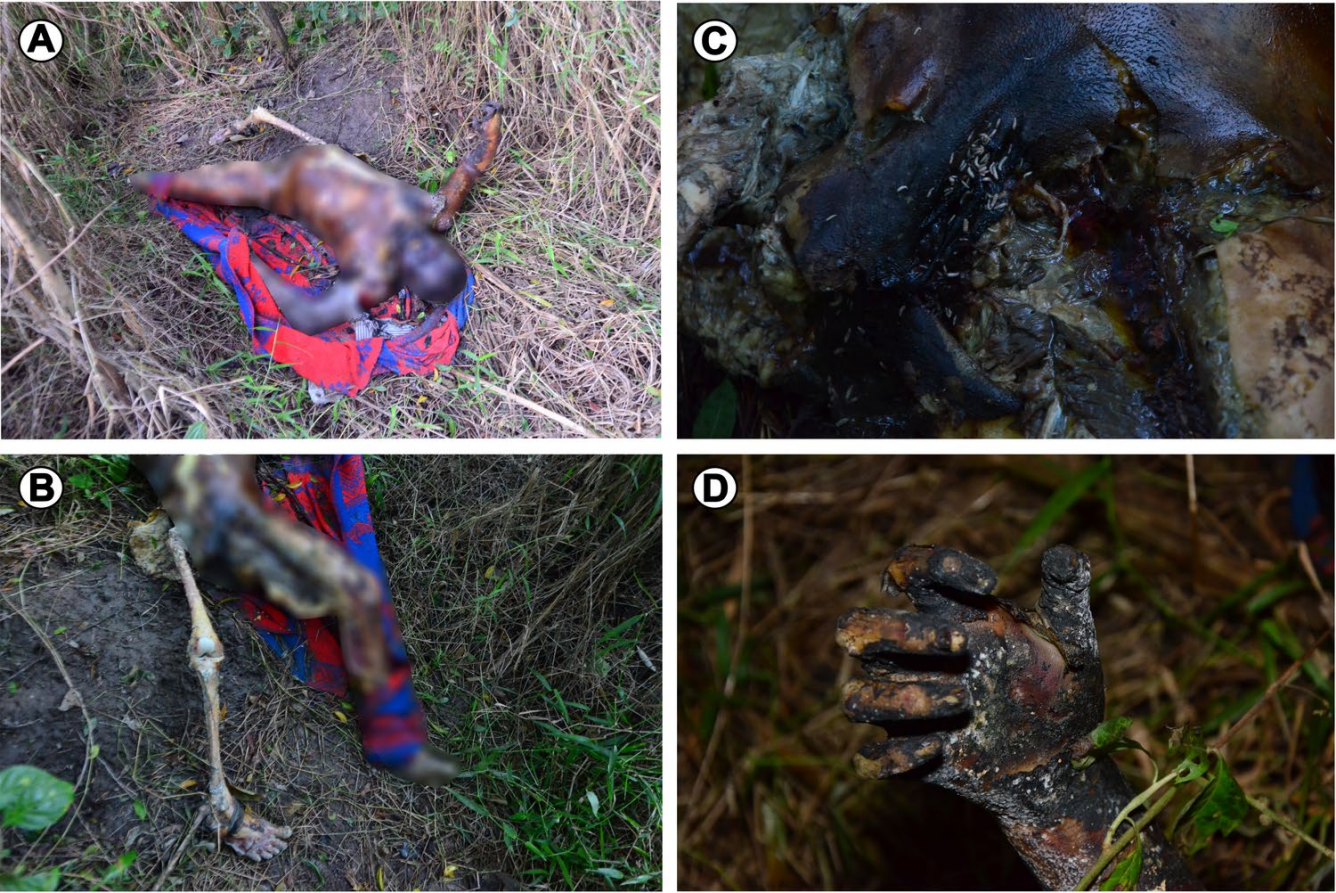
Crime scene. In: (**A**) the surroundings where the corpse was found, showing no signs of fire on the vegetation, blanket, or clothing; (**B**) in perspective, the femur and tibia bones of one leg were exposed entirely due to attacks by large scavenger vertebrates; (**C**) a large number of larvae on the upper back; (**D**) superficial charring on the hand

The corpse was in a dorsal decubitus position on a blanket on the ground, in an advanced stage of decomposition, with partially exposed viscera and a visibly large number of fly larvae. The femur and tibia bones of one leg were exposed entirely due to an attack by large scavenging vertebrates. Signs of superficial charring were observed, particularly on the skin of the hands and arms, as well as on the clothing. No signs of fire were observed in the vegetation surrounding the body, nor on the blanket that partially wrapped the victim, indicating that the corpse was possibly transported after death (Fig. 2).

**Fig. 3:**
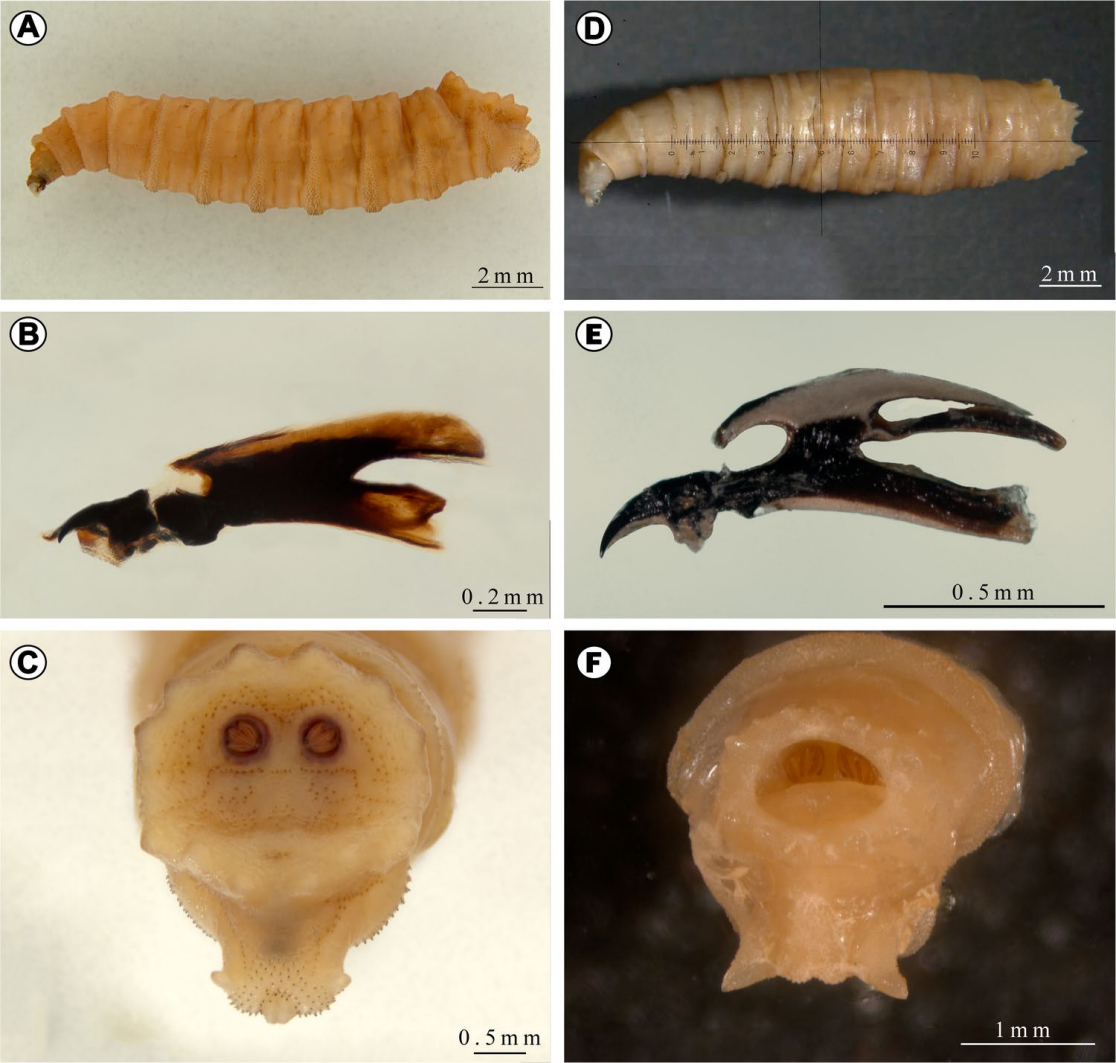
Third-instar larvae of *Hemilucilia segmentaria* (Calliphoridae) (**A**–**C**) and *Peckia* (*Euboettcheria*) *anguilla* (Sarcophagidae) (**D**–**F**) collected from the corpse and their diagnostic characters. In: (A) and (D) habitus; (B) and (E) cephaloskeleton; (C) and (F) anal division

Only fly larvae were found and collected on the corpse. Third-instar larvae collected were identified as belonging to the species *Hemilucilia segmentaria* (Fabricius) (Calliphoridae) and *Peckia* (*Euboettcheria*) *anguilla* (Curran & Walley) (Sarcophagidae) (Fig. 3). Local temperature records from the last 10 days, obtained from an automatic weather station, returned daily averages of approximately 15 °C. Morphometric data, such as weight (39.2 ± 0.5 mg; N = 10) and length (11.0 ± 0.2 mm; N = 10) for *H. segmentaria*, allowed us to infer that the total developmental time of this species, including egg incubation time at 15 °C (approximately 0.7 days or 18 h), was estimated of 9 days (= 222 h), based on the linear regression equations provided by Thyssen (2005):

$$equation[1]: y=0.1681x+1.9131$$

$$equation[2]: y=0.8038x-1.1236$$

where *y* indicates the development time in days and *x* is the predictor variable (weight, in mg, or length, in mm, in equations 1 and 2, respectively).

For *P*. (*E*.) *anguilla*, the total developmental time based on larval weight (62.5 ± 1.1 mg; N = 4) and length (14.3 ± 0.1 mm; N = 4) was estimated at approximately 6 days (= 154 h), from rearing data of laboratory-established specimens.

The PIA was estimated based on the age of the oldest insect collected from the corpse. Thus, the PIA allowed us to establish that the PMI minimum was approximately nine days. The police investigation reported that the victim had been missing since May 24th; the corpse was found on June 4th. Considering this scenario, entomological evidence was useful in determining that the death occurred close to the date of the disappearance.

## Discussion

The applicability of forensic entomology in legal matters depends on several factors that must be observed and followed systematically to prevent contamination or destruction of evidence, ensure the chain of custody, and guarantee the usefulness of the entomological samples (Amendt et al. 2007). One of these factors is the proper collection of entomological evidence, which can be conducted at the scene, during necropsy at the Medico-Legal Institute, or in both circumstances (Sanford 2015). Collection in both places, as performed in this study, enables a double-check examination for the oldest developmental insect stage present in the corpse.

As reported by Amendt et al. (2007), the PIA can accurately and reliably represent the PMI as long as it takes the total development time of the necrophagous insects, which are among the first colonizers, as a reference. This should be ensured through the following observations: (i) absence of barriers that delay or prevent the insects from reaching the corpse; (ii) absence of puparia and pupae at the crime scene (according to Sanford (2015), when present, these specimens are the oldest); and (iii) whether the corpse shows characteristic physical signs that it is in the early decomposition stages. In this case report, all of the conditions mentioned above were checked before using the PIA to estimate the PMI.

After overcoming the essential step of specific identification of the entomological evidence, information about its bionomy must be accessed to reach the PMI estimate (Catts and Goff 1992), including ensuring that it is not an accidental organism (see Smith 1986) in the context of the investigation being carried out. Besides, there are conditions that must be carefully observed for estimating PMI. First, observe the environmental local temperature and the preservation method used (Adams and Hall 2003; Matuszewski 2021). Then, choose the model for PMI estimation that best fits the sample: live or dead larvae. Temperature-dependent models, such as accumulated degree-day, need information of the total immature development or of a complete specific instar, which require rearing specimens in the laboratory (Thyssen and Oliveira-Costa 2024). Models that use morphometric data (weight or length) as dependent variables do not require immature rearing, although demand observation of the sample preservation conditions, i.e. larval stretch or not in heated water before fixation in ethanol, for example (Thyssen and Oliveira-Costa 2024).

In line with these issues, *H. segmentaria*, widely distributed in South and Central America, can be considered a good forensic indicator because it exhibits strictly necrophagous habits; additionally, it has been frequently reported to be associated with the wild environment, with few records of occurrence in wild-rural or wild-urban ecotones (Thyssen and Linhares 2007). Information about the bionomics, as well as the behavior of *H. segmentaria* relative to its habitat, was useful in corroborating the investigation's findings on the crime scene. Signs such as the absence of fire on the blanket and in the vegetation surrounding the body's location indicated that the corpse had been moved. However, a PIA obtained with a value close to the date of the victim's disappearance revealed that death occurred close to this date, the body's postmortem movement path was likely short, and that colonization had indeed begun at the site where the corpse was sighted.

*Peckia* (*E*.) *anguilla* is considered an omnivorous species and its attraction to food sources can range from those of animal (fish, bird, rat and mollusk carcasses, and feces) to plant-based sources, especially when fermented or in advanced decomposition stages (Madeira-Ott et al. 2022). It shows no preference for any particular environment, as evidenced by records in wild, rural, and urban areas (Madeira-Ott et al. 2022). The first record of *P*. (*E*.) *anguilla* reared on human corpses underscores the importance of taxonomic work in specific identification, particularly for immature stages, which have represented a major challenge for forensic science (Thyssen 2010; Prado et al. 2023). Furthermore, it highlights the need to expand knowledge about the bionomics of this species exposed to different temperatures to provide robust information for future PIA estimates based on this species.

Tissue moisture is a key factor for fly oviposition (Wardley 1921). Extensive charring can delay insect attraction and colonization, primarily due to elevated body temperature in the immediate post-burn period, the presence of smoke or fuel odors when accelerants are involved, and the rapid desiccation of tissues under such conditions (Wardley 1921; Anderson 2010; Oliveira-Costa et al. 2014). In contrast, moderate levels of burns, which cause skin cracks, especially on the abdomen, can contribute to accelerated insect colonization (Avila and Goff 1998). Thus, the colonization patterns observed in the case we report, associated with the massive number of larvae, indicate that charring was only superficial.

It is not uncommon to find vertebrate scavengers, such as mammals and birds, near corpses in the early stages of decomposition (Lira et al. 2020a), particularly in shaded areas (Anderson 2010). However, attention should be paid to the significance of taphonomic alterations caused by the action of these animals, which can range from a few punctures and skin ruptures to complete evisceration, on the cadaveric decomposition process (Anderson 2010; Lira et al. 2020a). In this case report, it was concluded that the PIA is reliably accurate, as even with a reasonably extensive removal of tissue from one of the victim's legs, the natural orifices where oviposition by pioneer flies is known to occur were not compromised.

The sequence of postmortem changes is often assessed to estimate PMI, although the most commonly evaluated phenomena — *algor mortis*, *rigor mortis*, and *livor mortis* — do not typically exceed 72 h (Campobasso et al. 2001). In turn, insects can reach and colonize the corpse within minutes of death, depending on access and environmental conditions, and may be present throughout the decomposition process (Catts and Goff 1992; Amendt et al. 2011). Considering the inherent limitations of cadaveric changes and the importance of obtaining reliable PMI estimates, entomological evidence should not be neglected, as unfortunately seen in most forensic institutions worldwide (Lutz et al. 2021). Furthermore, new strategies need to be devised to incorporate the collection and analysis of entomological evidence into routine forensic work to improve crime-solving rates.

## Data Availability

The datasets generated and analyzed during the current study are available from the corresponding author on reasonable request.
